# Role of Hydrogen Bonding in the Formation of Adenine Chains on Cu(110) Surfaces

**DOI:** 10.3390/ma9121016

**Published:** 2016-12-16

**Authors:** Lanxia Cheng

**Affiliations:** 1 EaStCHEM and School of Chemistry, University of St. Andrews, St. Andrews KY16 9ST, UK; lanxia.cheng@utdallas.edu; Tel.: +44-21-4507-3947; 2 Department of Material Sciences and Engineering, University of Texas at Dallas, Richardson, TX 75080, USA

**Keywords:** adenine, STM, DFT, Cu(110), hydrogen bonding

## Abstract

Understanding the adsorption properties of DNA bases on metal surfaces is fundamental for the rational control of surface functionalization leading to the realisation of biocompatible devices for biosensing applications, such as monitoring of particular parameters within bio-organic environments and drug delivery. In this study, the effects of deposition rate and substrate temperature on the adsorption behavior of adenine on Cu(110) surfaces have been investigated using scanning tunneling microscopy (STM) and density functional theory (DFT) modeling, with a focus on the characterization of the morphology of the adsorbed layers. STM results revealed the formation of one-dimensional linear chains and ladder-like chains parallel to the [110] direction, when dosing at a low deposition rate at room temperature, followed by annealing to 490 K. Two mirror related, well-ordered chiral domains oriented at ±55° with respect to the [110] direction are formed upon deposition on a substrate kept at 490 K. The molecular structures observed via STM are rationalized and qualitatively described on the basis of the DFT modeling. The observation of a variety of ad-layer structures influenced by deposition rate and substrate temperature indicates that dynamic processes and hydrogen bonding play an important role in the self-assembly of adenine on the Cu(110) surface.

## 1. Introduction

Molecular self-assembly on solid surfaces is a research topic of extensive experimental [1,2,3,4,5,6,7,8,9] and theoretical studies [10,11], owing to the promising applications in future organic-based nanoelectronics [1,12,13]. Organic molecules, including DNA and RNA bases, are capable of forming a range of nanostructures from well-ordered, two-dimensional (2D) molecular networks to one-dimensional rows upon deposition on a surface [1,2]. Investigations of molecule-molecule and molecule-surface interactions that govern the structural and electronic properties of the molecular nanostructures formed upon adsorption are relevant, not only to control surface functionalization for biocompatible and biosensor applications [12,13], but also to understand the complex biomolecule-surface interactions in general [14].

The adsorption of adenine on well-defined single crystal surfaces has been studied as a prototype example to understand DNA base-surface interactions. Unlike other DNA base molecules, such as guanine, thymine, and cytosine, the adenine molecular structure consists of only one functional group, i.e., an amino (–NH_2_) group, and a larger aromatic ring, which are favorable for flat-lying molecular orientation when interacting with substrates [5]. For example, on HOPG (0001) [15,16,17], Ag terminated Si(111) [18], Cu(111) [19,20], and Au(111) surfaces [21,22], adenine has been shown to form a variety of extended 2D hexagonal networks driven predominantly by intermolecular hydrogen bonding. Experimental measurements [18,20,22] and DFT calculations [23] both show that adenine adopts a flat-lying orientation in these supramolecular structures and the interaction with the substrate is weak. However, on Cu(110) [24,25,26] surfaces, since the first observation of one-dimensional chiral dimer chains aligning along the (1, ±2) directions by Qiao et al. [5], there has been some debate regarding the adsorption geometry and binding nature of adenine to copper. For instance, vibrational studies [5,27] and related theoretical calculations [11,25] indicate that adenine interacts relatively strongly with copper via the amino N atoms, and has its molecular plane oriented at ~26° away from the surface. Spectroscopic studies by Feyer et al. [28] and Bussolotti et al. [29] have reported a coverage dependent orientation transition from flat-lying to upright, in which both imino and amino N atoms participate in binding adenine to the copper substrate, therefore contributing to local charge transfer induced electrostatic interactions. Although these surface sensitive spectroscopic techniques are able to provide structural information on adenine ad-layer structures, they lack the direct imaging of surface adsorption structures provided by STM. A recent study also suggested that the most commonly observed chains growing along the (1, ±2) directions are composed of metastable dimers and chains formed by stable dimers grow along the (±4, 1) directions instead, because of the minimization of the repulsive van der Waals interactions between adjacent dimers [4]. Moreover, different adenine ad-layer structures, including double chains and hexagonal networks, have been shown to coexist and be influenced by the deposition rate on Cu(111) surfaces [30]. Therefore, an investigation of the effects of the preparation conditions is of considerable importance and may shed light on the debate on adenine adsorption geometry on Cu(110) surfaces as well as the fundamental understanding of the interaction between biomolecules and metal substrates.

Here, an investigation on the effects of annealing treatments up to 490 K on adenine overlayers on Cu(110), prepared by dosing at a low deposition rate with the substrate held at room temperature to achieve a medium coverage (θ ~ 0.60 monolayers, ML), is presented. A comparison is made with the deposition on a substrate maintained at 490 K. The features imaged via STM are compared with the geometries of various gas-phase dimers optimized via DFT calculations, allowing us to propose models for the observed structures.

## 2. Results and Discussion

### 2.1. Adenine Self-Organization at Low Deposition Rate

**Figure 1:**
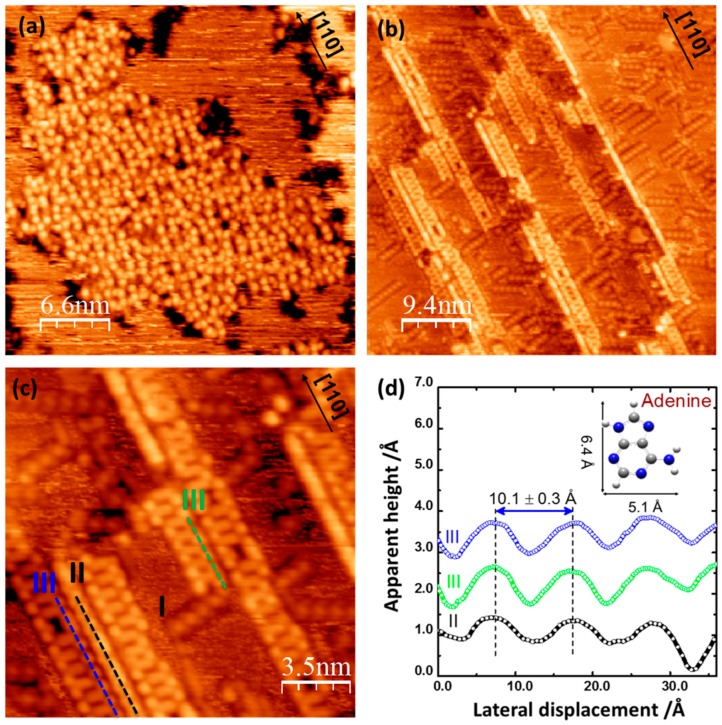
STM images of adenine ad-layer structures formed on the Cu(110) surface (~0.6 ML), after deposition and annealing to 490 K, at a deposition rate of ca. 0.018 ML/min. (**a**) (0.18 nA, −0.95 V, 33 × 33 nm^2^); (**b**) (0.143 nA, −1.1 V, 47 × 47 nm^2^); (**c**) STM image (0.145 nA, −1.14 V, 17 × 17 nm^2^) of the previously reported chiral I and the newly observed linear II and ladder-like chains III; (**d**) line profiles of chain structures II and III along the [110] direction; in the inset, the geometrically optimized adenine molecule with approximated dimension.

Figure 1a,b show STM images of adenine ad-layer structures obtained at a deposition rate of ~0.018 ML/min after deposition and following annealing to 490 K, respectively. Upon deposition, adenine molecules aggregate to form large disordered islands on the terraces (Figure 1a). Within the islands, adenine molecules appear as bright circular features seemingly interacting with surrounding molecules in a relatively random manner, although showing some partial ordering preferentially parallel to the [110] direction. At the island boundaries, some areas of dark contrast are seen; these are likely to represent adsorbate induced surface etching. The surface coverage of adenine is estimated to be about 0.6 ML. The formation of aggregated adenine islands is in contrast with the observation of some smaller distributed adenine clusters formed at the higher deposition rate of ~0.04 ML/min (Figure S1a), which then re-organized into chiral chains upon annealing to 490 K, similar to the structures previously reported [5]. However, after annealing to 490 K the preparations obtained at the slower deposition rate (Figure 1b), additional new molecular assemblies aligned along the [110] equivalent directions can be observed. Figure 1c shows an area where three types of molecular assemblies are identified and are referred as: chiral chains I, which are oriented along the (1, ±2) directions and essentially localized on terraces; single linear chains II, and ladder-like chains III, both found to condense along the substrate step edges, aligned along the [110] azimuth and commonly found adjacent to each other. These ordered adenine chain structures are thermally stable due to the strong adenine-substrate interactions (Figure S1), and our STM images suggest that the desorption of adenine starts upon annealing at 520 K, consistent with previous infrared studies by McNutt et al. [24].

Chiral chains I have been well-characterized and are commonly observed on a surface that is prepared by dosing at a relatively high rate and is then annealed to ca. 490 K. They consist of rows of dimers aligning along the (±1, 2) directions [4,5]. Linear chains II and ladder chains III have been observed here for the first time. Within a linear chain II, although individual intramolecular features are not readily resolved on the STM topographic image in Figure 1c, the periodicity along the [110] direction is estimated to be around 10.1 ± 0.5 Å (black line profile in Figure 1c,d) and the chain width is estimated to be 6.7 ± 0.5 Å. Because the footprint of an individual flat-lying adenine molecule can be approximated to its gas-phase dimensions based on the atomic van der Waals radii, ca. 6.4 × 5.1 Å (model in Figure 1d), these values indicate that adenine is likely to adsorb in a flat-lying or, at most, at a slightly tilted orientation with its short axis aligned almost parallel to the chain growth direction. Ladder chains III are composed of parallel chains with cross-linking rungs and have a width of 16.3 ± 0.5 Å (Figure 1c, blue line) and 14.5 ± 0.5 Å (Figure 1c, green line), respectively. The periodicity along the [110] direction is 10.1 ± 0.5 Å (Figure 1d, blue and green line profiles), which is nearly equal to four copper unit cells, indicating that the ladder-like chain may be commensurate with the substrate. Within each parallel row, the molecular features dimension along the [100] direction is 5.0 ± 0.5 Å. As this value is very close to the short dimension of an isolated adenine molecule, ~5.1 Å, it may indicate that adenine is likely to orient with its short axis aligned almost parallel to the [100] direction. Although the orientation of adenine molecules is thought to be different in chains II and III, the two chains have a similar periodicity of 10.1 ± 0.5 Å, as seen from the line profiles in Figure 1d. Additionally, the molecular features representing chains II and III appear to be brighter than those in the chiral chains I. This corresponds to a height increase of ca. 0.8 Å in comparison to that of the chiral chains I, in which adenine molecules are proposed to be lying flat on the metal surface with weaker π-metal interactions [22,31]. This increased height could be attributed to a tilted molecular orientation with respect to the copper substrate [11,26]. Nevertheless Near Edge X-ray Absorption Fine Structure (NEXAFS) measurement shows that the adenine molecular plane is nearly parallel to the Cu(110) surface [10].

In contrast to the chiral chains of structure I, both structures II and III are relatively less ordered and include some defects represented by individual molecules randomly missing within the chains. For structure III the defects often correspond to missing cross-linking rungs. In addition, two types of ladder chains can be observed, with one (indicated by the blue profile in Figure 1d) being ca. 1.8 Å wider than the other (green profile). This is tentatively ascribed to a different molecular orientation of the molecules constituting the rails of the ladder, as will be described in more detail in the DFT section (Figure 1c). The lengths of most of the ladder chains along the [110] direction is over 80 Å and longer than that of chiral chains I. The increased length may be favored by the formation of molecular structures commensurate with the substrate lattice, in addition to the existence of relatively stronger intermolecular hydrogen bonds that can contribute to the long range molecular ordering along the chain growth direction. This is consistent with the findings of Preuss et al. who considered both hydrogen bonding and molecular registry with the copper substrate as important factors to be accounted for in the formation of extended, long-range ordered molecular structures [26].

**Figure 2:**
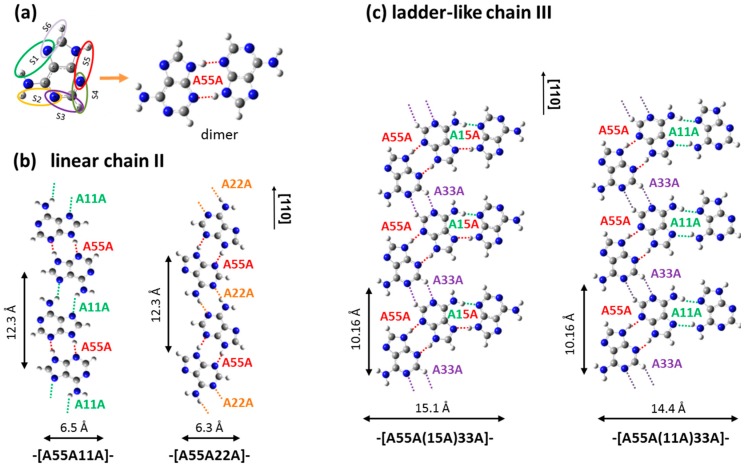
(**a**) Adenine molecular structure highlighting the six pairs of nearest N and H atoms referred to as binding sites S1–S6, and the most stable dimer, A55A, formed by connecting two molecules via binding site S5. Hydrogen bonds are color coded for ease of identification. Atom color scheme: H, light grey; C, dark grey; N, blue; (**b**) Model of linear chains II formed by connecting two adjacent A55A dimers via sites 1 or 2, labeled as chain -[A55A11A]- and -[A55A22A]- respectively; (**c**) Ladder chain III models formed by connecting -[A55A33A]- to an additional molecule through binding sites S5 or S1, giving rise to chains expressed as -[A55A(15A)33A]- and -[A55A(11A)33A]- respectively.

The appearance of chains II and III has some similarities with the hexagonal structures and parallel chains observed on a Cu(111) substrate [19,20] in terms of the chain width and lateral dimensions of each individual feature comprising the chains. However, the less ordered molecular chains formed on the Cu(110) surface are most likely related to the strong interaction between the amino N atom with the copper, plus the large Coulomb attraction originating from electron redistribution between adenine and substrate [5,25], which not only can cause a greater structure distortion than that predicted by the theoretical model, but also exert a more dominant force in anchoring the molecular registry on the copper atoms. Experimental results also found that the formation of linear and ladder chains along the [110] direction is most likely related to the disordered molecular aggregations, with an area of ~100 nm^2^, observed on copper terraces after deposition at room temperature and at a low deposition rate. Our STM observation of less ordered and short range adenine adlayer assemblies are also distinct from those ordered chain structures found with other nucleotides in terms of the molecule-substrate binding nature and molecular orientation. This is most likely caused by their different molecular structures. Since adenine only has one functional amino group that is available for anchoring the molecule to the substrate, and the existence of a more aromatic ring favors stronger π-substrate interactions [5], therefore, adenine tends to lie flat at most metal surfaces with H-bonding as the dominant inter-molecular interaction, whereas other nucleobases such as cytosine and guanine have both oxo and amino groups that tend to interact with copper more strongly, which lead to almost upright-molecular orientations with the formation of ordered molecular assemblies driven by dispersion interactions [32]. DFT calculations of possible adenine dimers, in terms of stabilization energy and optimized geometry, are helpful in the interpretation of the observed chains. In fact, each adenine molecule has six pairs of nearest N and H atoms which are referred to as binding sites S1–S6, as shown in Figure 2a. A binding site belonging to one adenine molecule can interact via double hydrogen bonds with a binding site on a neighbouring molecule to form an adenine dimer. Table 1 summarises gas-phase dimerization energies, **∆***E_dim_*, which are defined as the total energy of the relaxed dimer minus the total energy of two adenine molecules relaxed separately. Dimer geometries and stabilization energies, **∆***E_dim_*, are reported in Figure S2.

The formation of adenine dimers has been studied by several authors [5,26,33], as the dimers are regarded as the main building blocks for the construction of the ad-layers structures. Dimerization energies **∆***E_dim_* calculated in this work follow a similar trend of those previously reported [5,26,33], and identify A55A (Figure 2a) as the most stable dimer, with a stabilization energy of −1.02 eV. Figure 2b shows the two possible structural models for a one-dimensional linear chain II aligned along the [110] direction. These chains are formed by connecting two A55A dimers through binding site 1 or 2, to develop into a one-dimensional homochiral chain, denoted as -[A55A11A]- or -[A55A22A]-. According to our DFT calculations, the gas-phase stabilization energies of hydrogen bonded dimers A11A and A22A are −0.62 eV and −0.72 eV, respectively, therefore a chain based on the -[A55A22A]- monomer is likely to be more stable than one based on -[A55A11A]-. Gas-phase optimization, however, does not account for the effects of the registry of the chain on the substrate, which could play an important role in stabilizing the final structure. The lateral widths of the constructed homochiral chains for each model are ~6.5 Å, which is in good agreement with the measured lateral chain dimension of 6.7 ± 0.5 Å observed via STM. Preuss et al. [26] have calculated the energetics of various chains adsorbed in the registry on the Cu(110) surface and found that one dimensional chains based on -[A22A]- and -[A55A]- are more favorable in terms of their adsorption energies and stabilization energies. This is in line with the present findings. The structural model proposed here is also consistent with similar molecular features observed via STM by Furukawa et al. [30].

To describe the ladder-like chains of structure III, a number of different geometries were considered, in order to construct the structural model with the best fit molecular geometries observed in STM. The models shown in Figure 2c were considered as the closest match to the observed structures. In these models, two homochiral dimers A55A are connected through site 3 to yield a chain based on a -[A55A33A]- unit, with a periodicity of 10.16 Å along the chain growth direction. This value is close to the value of 10.2 ± 0.5 Å measured via STM shown in Figure 1c,d and, as already highlighted, nearly equal to four copper unit cells, strongly indicating that the chain may be commensurate with the substrate. A33A has a stabilization energy of about −0.16 eV, therefore it has a small contribution in the stability of the total system, and this could account for the random missing molecule in this position. Nevertheless, a strong stabilization effect may be exerted by the commensurability of the chain with the substrate. To complete the description of the ladder-like chains, a further adenine molecule has to be included. This has two coordination possibilities through hydrogen bonding, namely A11A and A15A. The stabilization energies of dimers A15A and A11A are −0.82 eV and −0.62 eV, respectively. The inclusion of these two dimers yields two calculated widths of 15.1 Å and 14.4 Å for ladders -[A55A(15A)33A]- and -[A55A(11A)33A]-, respectively. The wider ladder chain dimension is approximately 1.0 Å narrower than the corresponding measured value, ca. 16.3 Å; however, this small difference might be induced by structural relaxation upon adsorption, since the periodic potential well of the Cu(110) surface can constrain the molecular arrangement in the process of molecular registry on specific sites [25]. In fact, it is reported that an increase in the intermolecular distance with respect to the values calculated for gas-phase models by up to ~0.5 Å [18] may occur because of the interaction with the substrate. Preuss et al. [26] also found an increase in the length of the intermolecular distance upon interaction with the Cu(110) lattice. STM images show a higher number of wider chains, based on -[A33A(15A)55A]-, than narrower chains, based on -[A33A(11A)55A]-, which are more likely due to the inclusion of the relatively more energetically favorable dimer of A15A.

### 2.2. Chiral Domains Formed on a Substrate Held at 490 K

**Figure 3:**
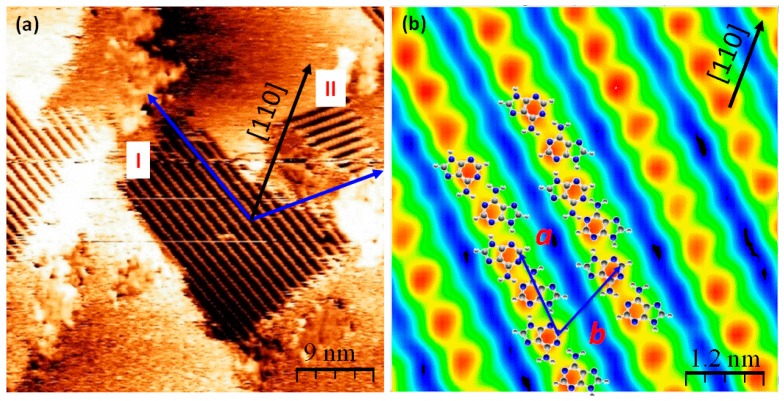
STM image of adenine chiral domains I and II formed on a Cu(110) surface maintained at 490 K. (**a**) (0.61 nA, −1.2 V, 45 × 45 nm^2^); (**b**) Magnification of the adenine molecular structure in domain I (0.68 nA, −1.1 V, 5.8 × 5.8 nm^2^) with superposed models consisting of adenine chains based on the -[A55A22A]- unit. The red sphere refers to single adenine molecular feature composed of the chiral chains; Vector *a* refers to the inter-molecular periodicity, and *b* is the inter-chain separation observed within the adenine structures in domain I.

When adenine is dosed on the substrate maintained at ca. 490 K, two distinct domains composed of ordered adenine rows are observed, labelled as I and II in Figure 3a. Previous studies of the adsorption of adenine are commonly carried out at room temperature and followed by annealing to form ordered adenine chain structures. However, the dosage of adenine at higher substrate temperatures is also fundamentally interesting to help understand the molecule-substrate interactions within adenine adlayers at a low thermal barrier surface.

Since the adsorption of pro-chiral molecules on surfaces typically produces equal amounts of each enantiomer, if there is chiral segregation, mirror related domains are formed. Each domain consists of adenine molecules of the same chirality. Here, the angle between the chain growth directions in the two domains is approximately 110°. Each domain is orientated at 55° with respect to the [110] axis of the substrate, indicated with a black arrow in Figure 3a.

A magnified STM image of domain I is shown in Figure 3b. Within this domain, there exists only one type of feature, the dimensions of which are 4.4 Å by 7.2 Å; this size matches the approximate footprint of a single flat lying or slightly tilted adenine molecule. Therefore, each of the features observed in the domain is assigned to a single adsorbed adenine molecule. The periodicity of the molecules along the chain is *a* = 12.8 ± 0.5 Å, and in the other direction is *b* = 11.2 ± 0.5 Å. The unit cell vectors are not aligned along the high symmetry directions of the substrate and so the unit cell is assigned a *C*_2_ symmetry; this finding is consistent with the observation of two mirror related domains. The rhombic unit cell shape and periodicities of the overlayer structures are also shown in Figure 3b, and the angle between the two unit cell vectors is 70° ± 2°.

A proposed model overlaid on the electron density map of the ordered structure in domain I is shown in Figure 3b. Along the rows, the chains are formed by alternation of the hydrogen bonded pairs A55A and A22A and the resulting rows are homochiral. The stabilization energy of dimer A55A is −1.02 eV as already highlighted; this is the basic unit employed to construct the models for most of the observed adenine structures. Dimer A22A has a stabilization energy of −0.72 eV; therefore, it is less stable than dimer A55A. Both A55A and A22A dimers have the nitrogen atoms participating in the formation of hydrogen bonds, therefore the proposed isolated units -[A55A22A]- are considered as energetically favorable for the construction of a model that agrees well with the features observed in the STM images. According to the DFT geometrical optimization, the periodicity along the adenine rows is 12.29 Å and is determined by the hydrogen bonding. The intermolecular distance is 6.14 Å, consistent with the length of vector *a* determined from the periodicity of the ad-layer features in the two domains. In each row, adjacent molecules are stabilized by intermolecular hydrogen bonds that dictate the growth direction; the proposed molecular arrangements are in good agreement with the features observed via STM. Additionally, the hydrogen bonding interaction of molecules in adjacent rows is excluded, as indicated by the large inter-row separation. In fact, the periodicity along vector *b* is more likely to arise from a combination of van der Waals interactions between adjacent chains [4] and strong substrate-adsorbate interaction [26].

**Figure 4:**
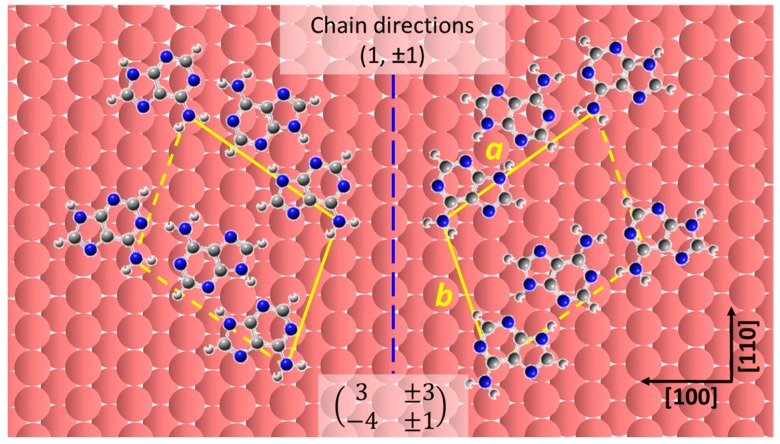
Proposed molecular registry of adenine on the Cu(110) substrate: one amino and one imino N atom of each adsorbed molecule are located at on-top positions in order to maximize the molecule-substrate interactions. The two domains have opposite chirality and are related by the mirror plane aligned along the [110] direction (blue dashed line). The red sphere represents the Cu atoms arranged in Cu(110) crystal lattice; Vector *a* refers to the inter-molecular periodicity, and *b* is the inter-chain separation within the proposed adenine structures in adenine chiral domains.

The rationality of the proposed model is further confirmed by the registry of the model over the Cu(110) lattice. As shown in Figure 4, two mirror related domains are produced, each consisting of adsorbed species of the same chirality.

The structures can be described in matrix notation as $\left(\begin{array}{cc} 3 & \pm 3\\ -4 & \pm 1 \end{array}\right)$ with a unit cell of 139 Å^2^ containing one adenine dimer. The growth direction of each adenine row is closely related to its chirality; molecules of opposite chirality are arranged at an angle of ±55° with respect to the [110] direction and grow along the (1, ±1) directions; this is consistent with the experimental results mentioned above. Given the proposed registry, the imino N–Cu interactions are also significantly facilitated by accommodating the two imino N atoms on each side of the amino group closely to the on-top sites, in addition to the amino N–Cu on-top interaction indicated by previous theoretical calculations [5,25]. Density functional Theory-Generalized Gradient approximations (DFT-GGA) calculations by E. Rauls et al. [11] predict that the orientation where the molecules bind via two imino N atoms to the substrate in the same way as in our proposed model is more energetically favorable than the interaction taking place between the amino N atom and copper atoms.

Therefore, we tentatively propose that the observed large two-dimensional ordered domains originate from more than one pair of N–Cu interactions, particularly along vector *b*. This is mainly derived from the large inter-row distance, 10.8 Å, estimated from the proposed model, which is in good agreement with the measured values. Along vector *a*, the balance between short range double hydrogen bond interactions and strong substrate-adsorbate interactions leads to the formation of longer chains.

## 3. Materials and Methods

All experiments were performed in an ultra-high vacuum (UHV) system equipped with an Omicron variable temperature scanning tunneling microscope (VT-STM), Ar ion sputtering and annealing facilities, a quadrupole mass spectrometer, and low energy electron diffraction (LEED). A clean Cu(110) crystal surface was obtained by cycles of argon ion sputtering (typically 800 V, 1.5 × 10^−5^ mbar, 20 μA) followed by annealing to 800 K; surface cleanliness was assessed by the appearance of a sharp (1 × 1) LEED pattern with low diffuse background and flat terraces on STM. Adenine (99% purity, Sigma-Aldrich Ltd., Gillingham, UK) crystalline solid was dosed on the clean Cu(110) substrate by thermal sublimation at 313 K from a home-built evaporator. The sublimation temperature remained constant during deposition and was monitored via a K-type thermocouple sensor to ensure reproducibility. The deposition rate employed (~0.018 ML/min) is about half of what was used by Qiao et al. [5,34] to obtain chiral, one-dimensional adenine dimeric chains. The deposition rate was evaluated by noting the deposition time taken to obtain a specific coverage; monolayer coverage refers to the surface fully saturated by one monolayer of adenine. The background pressure in the chamber was typically below 1 × 10^−10^ mbar and increased to ca. 1 × 10^−9^ mbar during deposition. All STM measurements were performed in UHV at a base pressure of ca. 1 × 10^−^^10^ mbar and at room temperature. STM images were collected in constant current mode using electrochemically etched tungsten tips and were processed using the WSxM 4.0 software package [35]. Proposed adenine gas-phase dimers were geometrically optimized using the Gaussian-03 software package [36] with the 6-31G basis set using hybrid density functional theory (DFT) with the non-local Becke’s three parameter functional (B3LYP) [37].

## 4. Conclusions

Adenine self-assembly on the Cu(110) surface has been investigated using scanning tunneling microscopy under different preparation conditions and the resulting structures were modeled via gas phase DFT calculations. Both the deposition rate and the annealing temperature, as well as the coverage, were found to influence the formation of adenine ad-structures. At a low deposition rate (ca. 0.018 ML/min) and medium coverage (~0.6 ML), adenine structures evolved from disordered islands to double chains with increasing annealing temperature, up to ca. 430 K. Increasing the annealing temperature up to ca. 490 K leads to the formation of ordered linear and ladder-like chain structures along the [110] direction, in addition to the previously reported chiral chains oriented along the (1, ±2) directions. Ordered mirror related domains were created when depositing adenine onto the substrate maintained at 490 K. For all the observed ad-layer structures, models based on connecting the stable dimer A55A via a variety of hydrogen bonding sites have been proposed. The formation of different chains after thermal treatments is thought to be closely related to the initial molecular aggregation achieved at a low deposition rate. The preferred registry and alignment of the stable dimer A55A with respect to the substrate upon annealing determines the orientation according to which the other dimers connect, via directional hydrogen bonds. This results in the formation of different chain structures. Upon deposition at an elevated substrate temperature, 490 K, substrate-adsorbate interactions, originating from the interaction of the amino N atom with copper and a partial contribution from imino N–Cu interactions, account for the formation of the observed well-ordered two-dimensional domains. This study provides an insight into the complex scenario of the interactions occurring between adenine molecules and the Cu(110) substrate.

## Figures and Tables

**Table 1:** Gas-phase adenine dimerization energies, **∆***E_dim_*/eV.

Dimer Type	A55A	A22A	A11A	A66A	A44A	A33A	A15A
This work	−1.02	−0.72	−0.62	−0.27	−0.16	−0.16	−0.82
Reference [5]	−0.44	−0.19	−0.27	−0.03	−0.03	−0.02	–
Reference [26]	−0.85	−0.77	–	−0.17	–	−0.08	–
Reference [33]	−0.79	−0.6	−0.49	−0.18	−0.1	−0.09	–

## Supplementary Materials

The following are available online at www.mdpi.com/1996-1944/9/12/1016/s1. Figure S1: STM images of adenine chiral chains obtained at a faster deposition rate and proposed structural model; Figure S2: Gas phase adenine dimers DFT optimized; Table S1: Stabilization energy per molecule of adenine *n-mer* species. Gaussian 03 software package full reference.
