# Supplementary material for: Role of Hydrogen Bonding in the Formation of Adenine Chains on Cu(110) Surfaces
Source: Materials (Basel). 2016 Dec 16;9(12):1016. doi: 10.3390/ma9121016 (PMC5457020; doi:10.3390/ma9121016)
Supplement: Supplementary file 1 [file materials-09-01016-s001.pdf]

Supplementary Materials: Role of Hydrogen Bonding in the Formation of Adenine Chains on Cu(110) Surfaces

Lanxia Cheng

When deposition is performed at a deposition rate of ~0.041 ML/min and at room temperature, adenine molecules aggregate into small islands and short molecular arrays on the copper terraces, as shown in Figure S1a. Figure S1b,c show ordered adenine dimer chains aligning along the (±1, 2) directions, obtained after annealing to 490 K, in agreement with that reported by Qiao et al. [1] and Zhang et al. [2]. A model of these chains is proposed in Figure S1d.

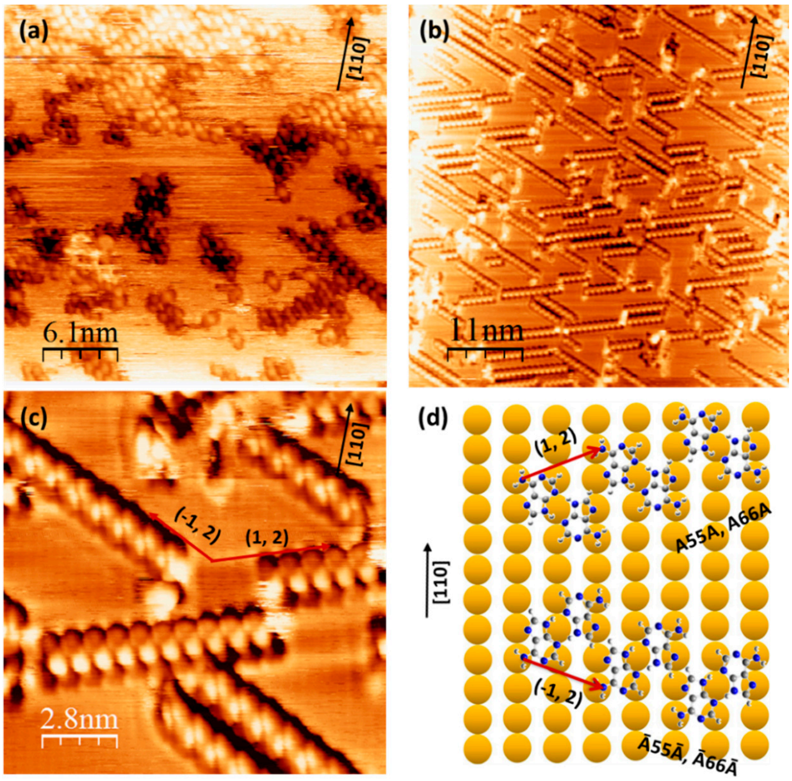

Figure S1. STM images of adenine adsorbed on Cu(110) surfaces showing the evolution of the superstructures as a function of the annealing temperature. (**a**) Room temperature (0.314 nA, −1.2 V, 30 × 30 nm^2); (**b**,**c**) annealing to 490 K (0.51 nA, −1.15 V, 55 × 55 nm^2 and 14 × 14 nm^2); (**d**) Proposed model of adenine chiral chains adsorbed in the registry on the Cu(110) substrate. $\bar{\mathrm{A}}$ indicates an enantiomer of A, obtained by reflecting A on a mirror plane normal to the page.

Figure S2 shows the six binding sites for adenine and the possible centro-symmetric dimers discussed in the text, with respective dimerization energies, ΔE_{dim}.

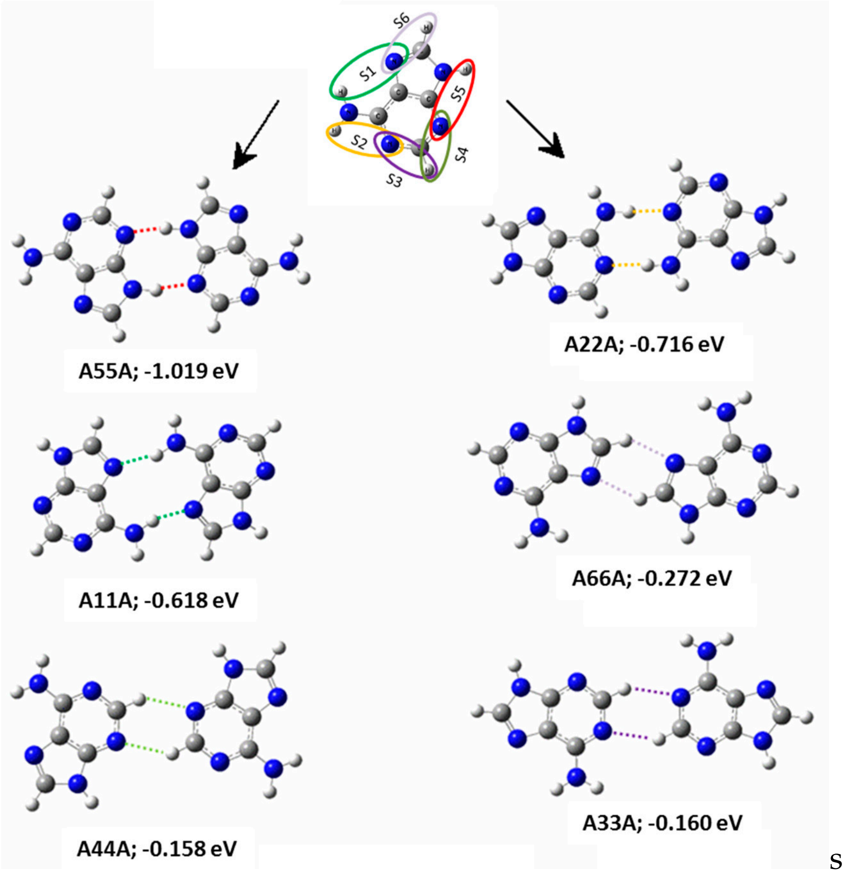

S

Figure S2. (**Top**) Adenine molecular configuration labeled with the six sites that can participate in the formation of hydrogen bonding between adenine molecules to form dimers; (**Bottom**) Six centro-symmetric adenine pairs and the corresponding stabilization energies are given. Proposed adenine gas-phase dimers were geometrically optimized using the Gaussian-03 software package [3] with the 6-31G basis set using the hybrid density functional theory (DFT) with the non-local Becke's three parameter functional (B3LYP) [4,5].

Table S1 reports stabilization energy per molecule of the adenine *n-mer* species calculated. $\Delta E_{n\text{-}mer}$ are calculated as:

$$\Delta E_{n-mer} = \frac{1}{n}\left(E_{n-mer,relaxed} - n \times E_{adenine,relaxed}\right)$$

where n is the number of molecules considered, $E_{n\text{-}mer,relaxed}$ is the energy of the relaxed *n-mer*, and $E_{adenine,relaxed}$ is the energy of a relaxed adenine molecule.

Table S1. Stabilization energy per molecule of adenine *n-mer* species.

n-mer	$E_{n\text{-}mer,relaxed}$/Hartree	$\Delta E_{n\text{-}mer}$/eV	$\Delta E_{n\text{-}mer}$ per Molecule/eV
A	−467.15988800	–	–
A55A	−934.35722133	−1.0189	**−0.5094**
A22A	−934.34608626	−0.7159	−0.3579
A11A	−934.34249141	−0.6181	−0.3090
A66A	−934.32976414	−0.2718	−0.1359
A44A	−934.32557915	−0.1579	−0.0789
A33A	−934.32567288	−0.1605	−0.0802
A15A	−934.34974199	−0.8154	−0.4077
A55A15A	−1401.54879459	−1.8812	**−0.6270**
A55A11A	−1401.54115235	−1.6732	−0.5577

References

1. Chen, Q.; Frankel, D.J.; Richardson, N.V. Self-assembly of adenine on Cu(110) surfaces. *Langmuir* **2002**, *18*, 3219–3225.
2. Zhang, Y.Y.; Wang, Y.-L.; Meng, L.; Zhang, S.B.; Gao, H.-J. Thermally controlled adenine dimer chain rotation on cu(110): The critical role of van der waals interactions. *J. Phys. Chem. C* **2014**, *118*, 6278–6282.
3. Frisch, M.J.; Trucks, G.W.; Schlegel, H.B.; Scuseria, G.E.; Robb, M.A.; Cheeseman, J.R.; Montgomery, J.A., Jr.; Vreven, T.; Kudin, K.N.; Burant, J.C.; et al. *Gaussian 03, Revision E. 01*; Gaussian, Inc.: Wallingford, CT, USA, 2004.
4. Becke, A.D. Density-functional exchange-energy approximation with correct asymptotic behavior. *Phys. Rev. A* **1988**, *38*, 3098–3100.
5. Lee, C.; Yang, W.; Parr, R.G. Development of the colle-salvetti correlation-energy formula into a functional of the electron density. *Phys. Rev. B* **1988**, *37*, 785–789.
